# Cervical Microbiome and Cytokine Profile at Various Stages of Cervical Cancer: A Pilot Study

**DOI:** 10.1371/journal.pone.0153274

**Published:** 2016-04-26

**Authors:** Astride Audirac-Chalifour, Kirvis Torres-Poveda, Margarita Bahena-Román, Juan Téllez-Sosa, Jesús Martínez-Barnetche, Bernardo Cortina-Ceballos, Guillermina López-Estrada, Karina Delgado-Romero, Ana I. Burguete-García, David Cantú, Alejandro García-Carrancá, Vicente Madrid-Marina

**Affiliations:** 1 Dirección de Infecciones Crónicas y Cáncer, Centro de Investigación sobre Enfermedades Infecciosas, Instituto Nacional de Salud Pública (INSP) (Chronic Infectious Diseases and Cancer Division, Center for Research on Infectious Diseases, National Institute of Public Health), Cuernavaca, Morelos, Mexico; 2 Private Health Center for Gynecology, Cuernavaca, Morelos, Mexico; 3 Centro de Atención para la Salud de la Mujer (CAPASAM) (Center for Women’s Health), Health Services of the State of Morelos, Cuernavaca, Mexico; 4 Division of Clinical Research, Instituto Nacional de Cancerología (INCan), SS, Mexico City, Mexico; 5 Division of Basic Research, Instituto Nacional de Cancerología (INCan), SS, Mexico City, Mexico; Istituto Nazionale Tumori, ITALY

## Abstract

Cervical cancer (CC) is caused by high-risk human papillomavirus persistence due to the immunosuppressive tumor microenvironment mediated by cytokines. Vaginal microbiota determines the presence of certain cytokines locally. We assessed the association between cervical microbiota diversity and the histopathological diagnosis of each stage of CC, and we evaluated mRNA cervical expression levels of IL-4, IL-6, IL-10, TGF-β1, TNF-α and IFN-γ across the histopathological diagnosis and specific bacterial clusters. We determined the cervical microbiota by high throughput sequencing of 16S rDNA amplicons and classified it in community state types (CST). Mean difference analyses between alpha-diversity and histopathological diagnosis were carried out, as well as a β-diversity analysis within the histological diagnosis. Cervical cytokine mRNA expression was analyzed across the CSTs and the histopathological diagnoses. We found a significant difference in microbiota's diversity in NCL-HPV negative women vs those with squamous intraepithelial lesions (SIL) and CC(p = 0.006, p = 0.036).When β-diversity was evaluated, the CC samples showed the highest variation within groups (p<0.0006) and the largest distance compared to NCL-HPV negative ones (p<0.00001). The predominant bacteria in women with normal cytology were *L*. *crispatus* and *L*. *iners*, whereas for SIL, it was *Sneathia spp*. and for CC, *Fusobacterium spp*. We found higher median cervical levels of IL-4 and TGF-β1 mRNA in the CST dominated by *Fusobacterium spp*. These results suggest that the cervical microbiota may be implicated in cervical cancer pathology. Further cohort studies are needed to validate these findings.

## Introduction

Cervical cancer (CC) is the fourth most common cancer in women and the seventh overall worldwide, with an estimated 485 000 new cases and 236 000 deaths in 2013. CC caused 6.9 million disability-adjusted life-years (DALYs) in 2013 [1], and it was the second most common cause of death by cancer among Mexican women in 2011 (10.4%) [2]. CC is caused by a persistent infection with high-risk human papillomavirus (HR-HPV). However, a HR-HPV infection is considered a necessary but not a sufficient cause for CC development [3]. Mechanical factors, like vaginal douching or sexual intercourse, and biological factors, like bacterial vaginosis (VB) [4, 5], or sexually transmitted infections (STIs) [6] alter the vaginal microenvironment and have been identified as cofactors in the persistence of an HPV infection [7].

The vast majority of HR-HPV infected women never develop CC because an adequate immune response is capable of controlling the infection and preventing its progression to precancerous lesion [8]. This suggests that additional factors act in conjunction with the HPV to influence the risk of CC development. So far, the following determinant co-factors have been associated to the appearance of CC: socio-environmental factors (cultural barriers, extreme poverty, poor sanitation areas, and limited access to health care) [9], epidemiological factors (multiparity, use of oral contraceptives for more than five years, multiple sexual partners and smoking) [10, 11], and genetic factors related to the host (polymorphisms in immune response genes, which determine a deficient immune response and local immunosuppression) [12– 14]. Therefore, CC is a complex multifactorial disease, and further research is needed to improve our understanding of its etiology.

It has been recently proposed that abnormal vaginal microbiota plays an important role in the development of cervical neoplasm [15]. An epidemiologic study identified *Chlamydia trachomatis* as a co-factor for CC development [16]. Similarly, other studies show that some bacterial species seem to be associated with the development of other cancers, such as colon cancer; these studies also suggest that various bacterial species preferentially inhabit tumor sites [17, 18].

There are still several gaps in knowledge regarding the association between vaginal and cervical microbiomes and CC development [19]. Bacterial culture-based evidence indicates that some potential pro-oncogenic pathogens, which may be members of commensal microbiota, contribute to tumor initiation and development [20, 21].

CC is a long-standing disease, and there are stages previous to it during which the conditions of the cervical and vaginal environment are modified, including vaginal acidity and cytokine pattern that lead to a local immunosuppression state. The presence of Lactobacilli, a low vaginal pH (< 4.5) and antimicrobial peptides are part of the defense mechanisms present in the vaginal microenvironment [22]. When an imbalance of the defense system occurs, physicochemical changes arise and produce histological alterations of the vaginal mucosa and the cervical epithelium, all of which conditions a selective pressure on the microbiota [23]. In a CC microenvironment, the presence of immunosuppressive cytokines (TGF-ß1, IL-10) favors the persistenceof the HPV infection [24, 25, 26, 27]. Most studies of the female genital tract microbiome have been carried out at the vaginal level and few studies involve the cervical microbiota and cytokine profiles. A comparative genomic analysis of the vaginal microbiome has identified changes in microbiota diversity among women with genital human immunodeficiency virus (HIV) infection, with or without bacterial vaginosis (BV) [28] and in pregnant women [29]. Additionally, it has been proposed that the vaginal microbial ecosystem and the cytokine profile play a role in promoting cervical dysplasia [30], given that an abnormal vaginal microbiota has been associated with the acquisition of an HPV infection [31]. However, few studies have been carried out on the cervical microbiome as a modifier of the HPV natural history with respect to the development of cervical lesions and cervical neoplasm[32].

Considering that it has been proved that specific species of cervicovaginal microbiota modulate the inflammatory immune response in the female genital tract of healthy black South African women [33], it is possible that the cervical microbiome is involved in promoting the expression of immunosuppressive cytokines [34].We have hypothesized that the HPV infection and the development of SIL and CC are associated with changes in microbiota diversity and with cytokine expression patterns at the cervical level. Our study examined the association between cervical microbiota diversity and composition, according to an histopathological diagnosis of each stage of the natural history of CC, and the cervical expression levels of IL-4, IL-6, IL-10, TGF-β1, TNF-α and IFN-γ mRNA.

## Material and Methods

### Study design and population

A cross-sectional study was carried out, using samples from a biological bank built between 2008 and 2011 from newly diagnosed cases of squamous intraepithelial lesions (SIL) (n = 268 HPV-positive samples); women with a negative Papanicolaou and a normal colposcopy as controls (n = 205, 81 HPV-negative and 124 HPV-positive samples), recruited from the Women’s Health care Center in the State of Morelos (*Centro de Atención para la Salud de la Mujer del Estado de Morelos*) in Mexico between June 2008 and November 2011, and from cervical squamous cell carcinoma cases (n = 171 HPV-positive samples), recruited from the Gynaecology Service at the National Cancer Institute (INCan) in Mexico City, between September 2010 and December 2011. The Bioethics and Research Committees at INCan (reference number. INCan/CC/326/10CB/609) and at the National Institute of Public Health (*Instituto Nacional de Salud Pública*–INSP; reference number: CI814) approved the baseline study during which the biological bank was built. In addition, all participants gave their informed consent to use their biological samples for further research studies. Every subject was interviewed for lifestyle, socio-demographic and reproductive factors known to be associated with increased risk of CC [12].

For methodological convenience, we selected 32 cases for this study, non-cervical lesions (NCL: n = 10 HPV-negative; n = 10 HPV-positive), SILs (n = 4 HPV-positive) and CC (n = 8 HPV-positive). Inclusion criteria for the subsample selection related to the medical history were: patient´s recruitment on the same day of menstrual period (seven postretirement menstrual period days), the non-use of douches and no sexual activity in previous days of the sampling. Also, not having records of antibiotic or antifungal use in the last 30 days previous to sampling. Other inclusion criteria included: molecular HPV + diagnosis; DNA and RNA purity and integrity suitable for sequencing and quantifying mRNA by qRT-PCR, and being Mexican with Mexican parents and grandparents to ensure similar ancestry. The only exclusion criterion was not having sufficient reads. “We considered 1 000 as the threshold, after performing the bioinformatics sequence analysis. The studied population’s socio-demographic and reproductive-sexual characteristics are presented in **Table 1**. All of the study subjects were Mexican women whose age ranged from 22 to 61 years. There was a significant difference between the groups regarding the contraceptive method and the positive HPV test. CC patients reported no use of contraceptive methods more frequently than the NCL patients. Regarding the SIL cases, the most prevalent HPV genotypes were HR-HPV (non-HPV16 or 18), whereas among CC patients the most prevalent genotype was HPV 16.

**Table 1:** Analysis of reproductive/sexual lifestyle-related risk factors in the study population (cervical lesion and cervical cancer patients).

Characteristics	NCL/SIL/CC	
	N = 17/4/8	P value^&^
***Demographic***		
Ethnicity % Mexican mestizo	100/100/100	
Age (y)* Mean (SD)	34(8)/40(14)/43(11)	0.13
Age of menarche (y)* Mean (SD)	*13(1)/14(3)/13(3)*	0.77
***Behavioral***		
Age at first intercourse (y)* Mean (SD)	*17(1)/19(3)/17(4)*	0.23
*Parity+*		0.28
≤ 3	88.2/75/62.5	
> 3	11.8/25/37.5	
*Number of lifetime sexual partners+*		0.89
≤ 3	82.3/75/75	
> 3	17.6/25/25	
*Contraceptive method+*		**0.013**
None	6/0/50	
Non-hormonal	47/100/37.5	
Hormonal methods 6 months—5 years	47/0/12.5	
*Cancer family history+*		0.46
No	11.8/0/75	
Yes	88.2/100/25	
*Smoking history+*		0.32
No	82.4/50/62.5	
Yes	17.6/50/37.5	
***Biological/Clinical***		
*History of previous STDs+*		0.11
None	58.8/50/100	
Vaginosis	11.76/25/0	
Candidiasis	23.53/0/0	
Contagious mollusc	5.88/0/0	
HPV	0/25/0	
*Test for HPV infection*		**0.003**
Negative	41.18/0/0	
Other -HR-HPV	17.65/75/0	
HPV 18-positive	0/0/25	
HPV 16-positive	41.18/25/75	
*HSV-2 seroprevalence*		0.07
Positive	5.88/0/37.5	
Negative	94.12/100/62.5	

NCL: non-cervical lesion; SIL: squamous intraepithelial cervical lesion; CC: cervical cancer

HSV-2: Herpes simplex virus-2

The results are expressed as mean and SD for continuous variables. Categorical variables are expressed in percentages.

^&^Kruskal-Wallis for continuous variables* and Χ² for categorical variables+

Bold text denotes significant p values (p<0.05).

### Characteristics of the biological samples bank

Samples were taken from women’s cervix seven days after menses withdrawal. The biological bank used for this study is composed of DNA samples and cDNA extracted from cervical epithelial scraping swabs from women diagnosed with NCL and from fresh cell biopsies from women diagnosed with SIL and CC. Genomic DNA was extracted from cervical epithelial scrapings and biopsies previously digested with proteinase K, using the Genomic DNA Purification Kit (Fermentas Life Sciences, Vilnius, Lithuania). All possible measures were taken to avoid cross-contamination of samples during DNA extraction. DNA concentration and purity were evaluated by Thermo Scientific NanoDropTM 1000 Spectrophotometer (260/280) and DNA integrity was determined by electrophoresis in agarose gels at 1%. Total RNA was isolated from cervical samples using the Trizol reagent from Invitrogen. cDNA synthesis was carried out in the presence of 200 U of M-MLV reverse transcriptase and 2.5 ug of total RNA using standard conditions. The PCR reactions were performed in a reaction volume of 25 μL containing 1 μL of cDNA, 0.2 mM of dNTPs, 15 pmol of each primer, 2.5 μL of reaction buffer and1U of Taq DNA polymerase recombinant. Primers for the human housekeeping glyceraldehyde-3-phosphate dehydrogenase GAPDH (450 pb) were used to verify cDNA integrity.

Cervical specimens were previously tested for HPV. Viral DNA fragments from the samples were amplified by PCR using consensus primers MY09/MY11 [35], LIC1/LIC2 [36], and GP5/GP6 [37], which flank the L1 region of the HPV capsid. PCR amplification of GAPDH (556pb) was used as an internal control for DNA quality. Cell lines expressing HPV-16 (SiHa) and HPV-18 (HeLa) were used as positive controls. Deionized H_2_O served as a negative control. All products were analyzed by electrophoresis in acrylamide gels at 6%. Positive samples were visualized in an agarose gel at 1.5%. The DNA band obtained was extracted and purified with the MinElute Gel Extraction Kit (Qiagen, Hilden, Germany) and sequenced using the Sanger method. The HPV sequences were analyzed by BLAST. HPV was categorized according to its phylogenetic patterns into low- and high-risk types: HPV16 and HPV18. HPV status was confirmed with the Anyplex^TM^ II HPV HR Detection assay from Seegene^®^, based on multiplex real-time PCR, TOCE and DPO primer pairs technology, according to the supplier’s instructions. [38]

### Ethics statement

This study was conducted according to the principles expressed in the Declaration of Helsinki. It was approved by the Research, Ethics and Biosafety Committees at INSP (CI:1143). Written informed consent was obtained from all participants.

### Analysis of cervical cytokine mRNA expression

A qRT-PCR amplification was performed in duplicate for the gene expression analysis with the following TaqMan probes: GAPDH (ID-Hs99999905_mL), IL-4 (ID-Hs00174122_mL), IL-6 (ID-Hs00174131_mL), IL-10 (ID-Hs00961622_mL), TGF-β1 (ID-Hs00961622_mL), TNF-α (ID-Hs00174128_mL) and IFN-γ(ID-Hs00174143_mL). The amplification mix was prepared by adding 100 ng of each cDNA sample to a final reaction mixture of 10 μl containing 5μl of TaqMan PCR Master Mix for expression, 0.5 μl of probe and 3.5 μl of DNase-free molecular grade water. The amplification cycles(performed on a StepOnePlus™ from Applied Biosystems, Foster City, CA, USA) were as follows: 94°C for 10 minutes, 40 cycles at 94°C for one minute, 54°C for one minute, 72°C for one minute and 30 seconds, followed by 72°C for 15 minutes. GAPDH was used to normalize the amount of IL-4, IL-6, IL-10, TGF-β1, TNF-α and IFN-γ mRNA present in each sample [39]. Peripheral blood mononuclear cells stimulated with phytohemagglutinin for 72 hours were used to determine the dynamic range curves of IL-4, IL-6, IL-10, TGF-β1, TNF-α and IFN-γ expression; all standard curves were realized in triplicate. The expression level of mRNA for each cytokin estudied was calculated using relative quantification with the comparative Ct(2-ΔCt) method, taking GAPDH as the endogenous gene. Samples were analyzed in duplicate.

### High-throughput sequencing of 16S rDNA amplicons

Amplicons of ~456 bp containing V3-V4 variable regions from 16S rRNA genes were obtained for DNA libraries preparation. Primers 347F Forward 5'-GGAGGCAGCAGTRRGGAAT-3' and 803R Reverse 5'-CTACCRGGGTATCTAATCC-3', described by Nossa, were used [40]. A first, PCR was performed with the following conditions: 50 ng of template from each DNA sample were added to a final reaction mixture of 30 μl containing 1X reaction buffer, 2.5 mM MgSO_4_, 1mM dNTP, 0.2 U Platinum Taq DNA Polymerase High Fidelity (Invitrogen) and 5 pmol/μL of the primers. The amplification program was the following: 94°C for 3 minutes, then 30 cycles at 94°C for 30 seconds, 55°C for 30 seconds, 68°C for 30 seconds, followed by 68°C for 5 minutes. Amplicons were visualized by electrophoresis in agarose gel at 1%. The DNA bands obtained were purified by MinElute Gel Extraction Kit (Qiagen, Hilden, Germany).

Amplicons were re-amplified and processed with the same primers linked to adapter A of the 454 sequencing protocol followed by a 6-mer multiplex identifier and by the primer 347F. The reverse primer was the same for all the reactions linked to adapter B of the 454 sequencing protocol. The reaction conditions were as follows: 0.0625 ng of each DNA sample to a final reaction mixture of 30 μl containing 1X reaction buffer, 2.5 mM MgSO_4_, 1mM dNTP, 0.2 U Platinum Taq DNA Polymerase High Fidelity (Invitrogen) and 5 pmol/μL of primers 347F and 803R. Amplification cycles were the following: 94°C for 3 minutes, then 15 cycles at 94°C for 30 seconds, 55°C for 30 seconds, 68°C for 30 seconds, followed by 68°C for 5 minutes. Gel electrophoresis was performed in agarose gel at 1.5% to visualize the integrity of the amplified products. DNA fragments were purified by MinElute Gel Extraction Kit(Qiagen, Hilden, Germany) and afterwards quantified and assessed. Amplicons were pooled in eight libraries and mixed in an equimolar manner. Subsequently, the libraries were purified with Ampurebeads XP (Beckman Coulter, Inc). Emulsion PCR titration and yield calculation were then performed to determine the volume of beads to load into sequencing plates. Subsequently, massive sequencing was performed on the Genome Sequencer Titanium Roche-454 (AppliedScience) platform.

### Bioinformatics analysis

The raw sequences of the V3-V4 region from the 16SrRNA gene generated for each amplicon were processed with the Roche-454 complementary software, and *.sff files were generated. High quality reads were selected with the following criteria: reads with more than five consecutive bases with scores under 20 on the Qphred scale on a 30 bases moving window were discarded. Quality control (QC) was supervised with Thomas Girke’s R script that uses a fastq Quality.R library to plot the distribution of the rating assigned to each base according to its position on the Qphred logarithmic scale [41]. Once quality files and sequences were obtained separately from the *.sff raw files, demultiplexing was conducted in QIIME to identify the sequences belonging to each sample according to their molecular identifier (MID) [42].

The sequences were grouped into operational taxonomic units (OTUs) using the PyNAST algorithm for sequence alignment. All sequences which had a similarity of 99% or more were considered a single OTU [43]. A representative sequence of each OTU was chosen for later taxonomic identification. Taxa were assigned with the Uclust algorithm [44], using the Green Genes database (99% release in May 2013) as reference, so that any representative sequence aligned with a 99% similarity to a reference sequence would be assigned the same species name [45].

Sequences that did not align to the reference were extracted to another file for *de novo* assembly and to be considered in the diversity calculus. To determine if microbiota composition was able to cluster samples by diagnosis, an unsupervised hierarchical clustering based on the Bray Curtis dissimilarity between OTU abundance of each sample (**S1 Table**) was performed with R packages and plotted as a heatmap. Alpha diversity was described using a phylogenetic diversity (PD) whole tree and a Shannon diversity index (H´) calculation and their rarefaction. A distance matrix and principal components (PC) were calculated with UniFrac to define beta diversity [46]. Moreover, a coordinate analysis (PCoA) was graphed with QIIME scripts and visualized in Emperor [47].

We classified microbiota composition according to community state type (CST) based on the predominant taxa found in the samples. A cervical CST is a cluster of community states (species composition and abundance of a cervical community) that are similar in terms of the kinds and relative abundances of the observed phylotypes [48]. The clustering of community states was carried out by means of a hierarchical clustering based on the Bray Curtis dissimilarity between all pairs of community states and an average linkage.

### Statistical analysis

Relevant variables were compared between NCL vs SIL and NCL vs CC, using *χ*^2^ and Kruskal-Wallis test for categorical and continuous variables, respectively. T-student tests were carried out to determine the mean difference between the Shannon diversity index or the phylogenetic diversity whole tree and the histopathological diagnosis. Logistic regression models were used to determine the association between the histopathological diagnosis and the Shannon diversity index and the histopathological diagnosis (NCL independently of HPV status and SIL/CC) and PD whole tree, adjusting by age, parity, contraceptive method and HPV-genotype.

We estimated alpha diversity risk as an odds ratio (OR) with 95% confidence interval (CI). The estimated mean difference of bits units (Shannon diversity index) in cervix between SIL or CC and NCL independently of HPV status was evaluated by a linear regression analysis adjusting by age, contraceptive method and HPV-genotype. We evaluated the variation of weighted UniFrac distances using a Kruskal–Wallis test to check the beta diversity within each histopathological diagnosis group. A Wilcoxon Mann-Whitney test was carried out to evaluate the statistical significance of weighted UniFrac distances between SIL/CC vs NCL-HPV negative.

Cervical expression of IL-4, IL-6, IL-10, TGF-β1, TNF-α and IFN-γ mRNA was analyzed by histopathological diagnosis (NCL vs SIL and NCL vs CC) using the Wilcoxon Mann-Whitney test. Cervical expression of IL-4, IL-6, IL-10, TGF-β1, TNF-α and IFN-γ mRNA was analyzed across CST clusters by means of the Kruskall Wallis test. Finally, we performed direct correlation analyses between the Shannon diversity index and the cervical expression of IL-4, IL-6, IL-10, TGF-β1, TNF-α and IFN-γ mRNA. We performed all the statistical analyses using Stata statistical software, version 13.0 (StataCorp, Collage Station, TX, USA).

## Results

### General pattern of the cervical communities sampled

**Fig 1:**
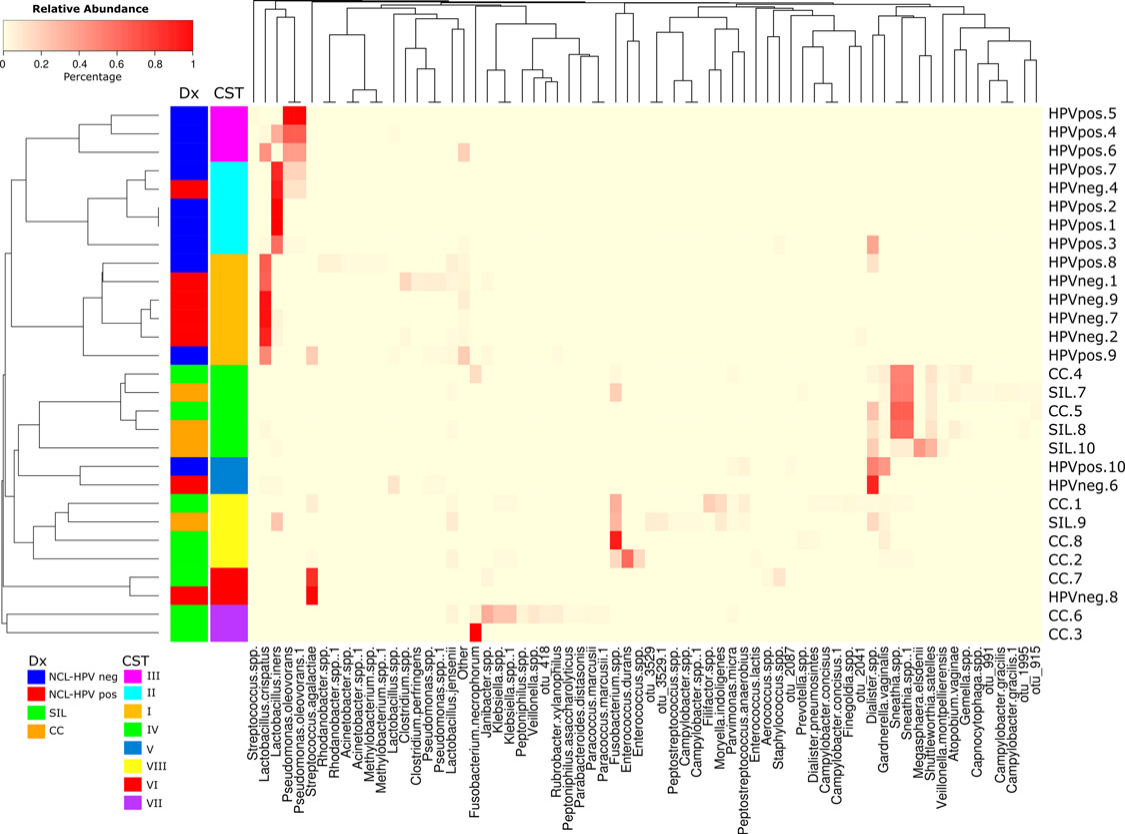
Community composition of cervical samples at the species level as determined by massively parallel sequencing on the 454 platform. Unsupervised heatmap of the relative abundance of microbial taxa found in the cervical microbial communities of 29 subjects, based on the Bray Curtis dissimilarity metric. The species present in relative abundance of 1% in at least one sample are listed on the X axis. The first bar on the left side represents the treatment as follows: red–HPV-negative without lesion; blue–HPV-positive without lesion; orange–squamous intraepithelial cervical lesion; green–cervical cancer. CST are depicted in the second left barside; pink–CST III, dominated by *Pseudomonas oleovorans*; cyan–CST II dominated by *L*. *iners*; orange–CST I dominated by *L*. *crispatus*; green–CST IV dominated by *Sneathia*; blue–CST V dominated by *G*. *vaginalis*; yellow–CST VIII dominated by *Fusobacterium spp*.; red–CST VI dominated by *S*. *agalactiae*, and purple–CST VII dominated by *Fusobacterium necrophorum*. Sample names appear on the right side of the graph. The cladograms at the top of the species names indicate the approximate evolutionary relationships between the species. CST: Community State Type. Dx: Histopathological diagnosis.

After QC, there were 311863 high-quality reads unevenly spread through the samples. To address this issue, 1000 random subsamples were obtained from the raw data of each sample, and they were used for further bioinformatics analysis. Alpha diversity rarefaction curves (**Fig A in S1 File**) show that after calculating H´in more than 400 reads, subsample diversity did not increase; therefore, 1000 sequences had sufficient sequencing depth for this study. On the unsupervised heatmap (**Fig 1**), samples from the NCL cluster together and independently of the HPV status, showed that HPV-negative women had a higher proportion of *Lactobacillus crispatus* (46%) and a smaller one of *Lactobacillus iners* (14.9%), whereas HPV-positive women had proportions of 13.3% and 2.1%, respectively. Interestingly, these proportions seemed to switch with the presence of HPV. Furthermore, *L*. *crispatus* was found in smaller proportions in SIL (14.4%) and CC (1.3%), whereas *L*. *iners* dropped to 2.1% in SIL and was not detected in CC.

Other species of *Lactobacillus*, *L*. *jensenii* and *L*. *vaginalis* were found only in samples from women with NCL. *Gardnerella vaginalis* was found mainly in HPV-negative women with NCL (11.5%), and it decreased gradually across the HPV-positive (6.9%), SIL (8.1%) and CC (3.3%) groups. *S*. *agalactiae* represented more than 90% of the microbiota composition of two of the samples. *Pseudomonas oleovorans* was observed in a relative abundance of 19% only among HPV-positive women with NCL. Bacteria from the *Fusobacteriales* order were found only in the SIL and CC groups. In the SIL group, *Fusobacterium spp*. displayed a relative abundance of 6.3%; *Sneathia spp*., 26.6%; *Shuttleworhia satelles*, 8.7%; and *Megasphaera elsdenii*, 10.4%; whereas the relative abundance of the same microorganisms in the CC group was as follows: 14%, 12.9%, 0%, and 2.2%, respectively. *Fusobacterium necrophorum* was only observed in the CC group (14.2%). This points to the fact that microbiota diversity and composition are different among the analyzed groups. To confirm this, alpha and beta diversity were analyzed.

**Fig 2:**
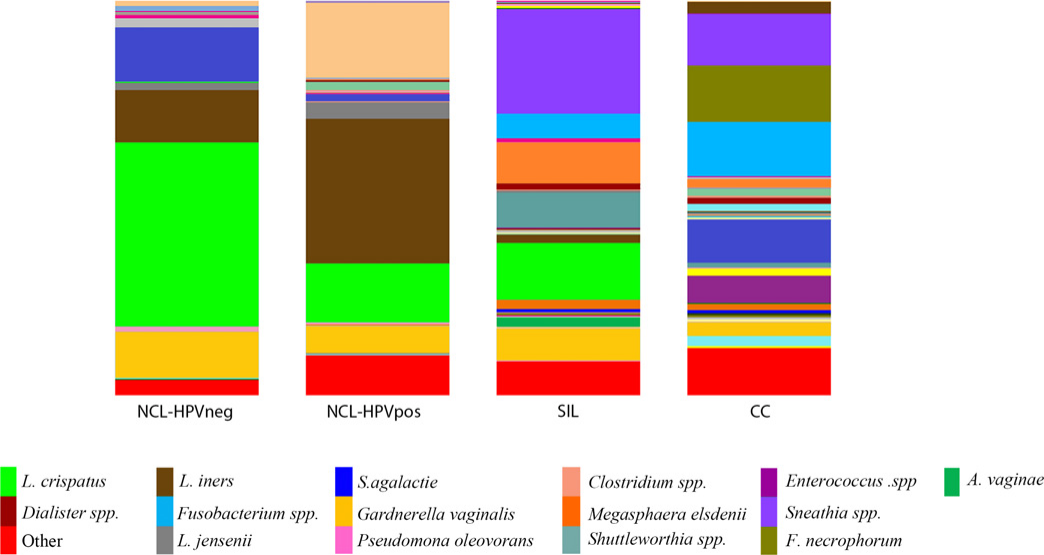
Community compositions according to histopathological diagnosis groups. Bar chart of relative abundance of species per group.

The cervical communities were classified into eight CSTs according to the dominant bacteria, as shown in **Table 2**. CST I isdominated by *L*. *crispatus* (21%), CST II by *L*. *iners* (17%), CST III by *Pseudomonas oleovorans* (10%), CST IV by *Sneathia spp*. (17%), CST V by *G*. *vaginalis* (7%), CST VI by *Streptococcus agalactiae* (7%), CST VII by *F*. *necrophorum* (7%), and CST VIII by *Fusobacterium spp*. (14%). Except for CST VI, all the samples were clustered according to the histopathological diagnosis. CST I was composed mainly of HPV-negative women with NCL; CST II and III were predominantly composed of HPV-positive women with NCL; CST IV was predominantly composed of SIL cases; CST V was composed mainly of women with NCL regardless of their HPV status; CST VIII was composed predominantly of CC cases, and CST VII included only cases of CC (**Fig 2**).

**Table 2:** Distribution of samples in each community state type (CST).

CST/Histopathological diagnosis	I (%)	II (%)	III (%)	IV (%)	V (%)	VI (%)	VII (%)	VIII (%)	Total
HPV-negative NCL	4(57)	1(14)	0	0	1(14)	1(14)	0	0	7
HPV-positive NCL	2(20)	4(40)	3(30)	0	1(10)	0	0	0	10
SIL	0	0	0	3(75)	0	0	0	1(25)	4
CC	0	0	0	2(25)	0	1(12)	2(25)	3(37)	8
Total	6 (21)	5 (17)	3 (10)	5 (17)	2 (7)	2 (7)	2 (7)	4 (14)	29

CST I dominated by *Lactobacillus crispatus*; CST II dominated by *Lactobacillus iners*; CST III dominated by *Pseudomonas oleovorans*; CST IV dominated by *Sneathia spp*.; CST V dominated by *Gardnerella vaginalis*; CST VI dominated by *Streptococcus agalactiae*; CST VII dominated by *Fusobacterium necrophorum*; CST VII dominated by *Fusobacterium spp*.

NCL: non-cervical lesion; SIL: squamous intraepithelial cervical lesions; CC: cervical cancer

### Comparison of cervical microbiota diversity across CC stages

#### Alpha diversity

**Fig 3:**
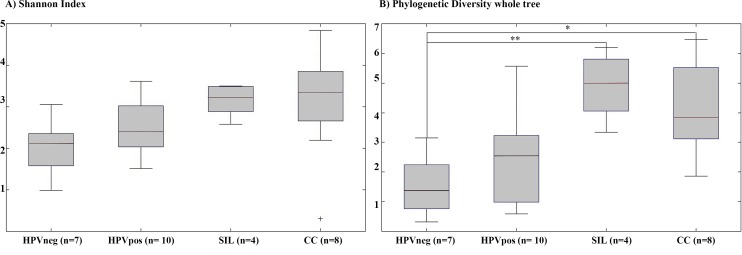
Comparison of the Shannon diversity index and the PD whole tree logistic according to the histopathological diagnosis group. The boxplots show the distribution of the H’ (bit units) and PD values across all the samples.

Even though rarefaction curves for the Shannon index (**Fig A in S1 File**) showed a higher diversity in the CC and SIL groups than among the HPV-negative and -positive women with NCL, we found that diversity per se, which accounts only for richness and relative abundance, is not sufficient to determine the stage of developmentof CC. Nevertheless, when it comes to indices that consider phylogenetic metrics as a PD whole tree, we found a significant difference regarding phylogenetic diversity between HPV-negative NCL and SIL and between HPV-negative NCL and CC (p values: 0.006 and 0.036, respectively) (**Table 3**). In other words, the composition of the cervical microbiota is different between groups. The box plots in **Fig 3**show the distribution of the Shannon diversity index and the PD whole tree across histopathological diagnosis groups. The Shannon diversity index shows an increasing trend but it is not significant. The PD whole tree shows a significant difference between HPV-negative NCL and SIL and between HPV-negative NCL and CC. SIL and CC groups display the most diverse cervical microbiota. We did not find an association between the histopathological diagnosis and the Shannon diversity index (NCL regardless of HPV status and SIL/CC), nor between the PD whole tree and the histopathological diagnosis according to the logistic regression analysis (**Table 4**). In assessing the association between the Shannon diversity index for the cervical microbiome samples and the histopathological diagnosis, the mean estimated difference of bit units between CC and NCL was 1.11 (95% CI, 0.057–2.165, (p = 0.04) (**Table 5**). This confirms that microbiota diversity in CC cases is higher than in the NCL group.

**Table 3:** Mean difference analysis between the Shannon diversity index or the phylogenetic diversity whole tree and the histopathological diagnosis.

Histopathological diagnosis		Shannon diversity index (H’)			PD whole tree		
Comparison group 1	Comparison group 2	Mean 1 (SD)	Mean 2 (SD)	P value^&^	Mean 1 (SD)	Mean 2 (SD)	P value^&^
HPV-neg NCL	HPV-pos NCL	2.00 (0.63)	2.49 (0.70)	1	1.55 (0.99)	2.49 (1.61)	1
HPV-neg NCL	SIL	2.00 (0.63)	3.14 (0.38)	0.18	1.55 (0.99)	4.88 (1.13)	**0.006**
HPV-neg NCL	CC	2.00 (0.63)	3.08 (1.28)	0.498	1.55 (0.99)	4.14 (1.49)	**0.036**
HPV-pos NCL	SIL	2.49 (0.70)	3.14 (0.38)	0.792	2.49 (1.61)	4.88 (1.13)	0.174
HPV-pos NCL	CC	2.49 (0.70)	3.08 (1.28)	1	2.49 (1.61)	4.14 (1.49)	0.318
SIL	CC	3.14 (0.38)	3.08 (1.28)	1	4.88 (1.13)	4.14 (1.49)	1

NCL: non-cervical lesion; SIL: squamous intraepithelial cervical lesions; CC: cervical cancer

PD: phylogenetic diversity

^&^ P value, Student’s t-test

Bold text denotes significant p values (p < 0.05).

**Table 4:** Association analysis between histopathological diagnosis and alpha diversity indexes.

Histopathological diagnosis	N	Shannon diversity index (H’)		PD whole tree	
		OR^a^ (95% CI)	p value*	ORa (95% CI)	p value*
NCL regardless of HPV status	17	1		1	
SIL/CC	12(4/8)	3.35(0.636–17.654)	0.15	3.30(0.756–14.485)	0.11

SIL: squamous intraepithelial cervical lesions; CC: cervical cancer

^a^ Odds ratio adjusted by age, contraceptive method and HPV-genotype.

*Bold text denotes significant p values (p<0.05)

**Table 5:** Estimated mean difference of bit units (Shannon diversity index) in cervix between NCL regardless of HPV status and SIL or CC.

Histopathological diagnosis	N	Shannon diversity index (H’)	
		ß^a^ (95% CI)	p-value*
**SIL**	17	0.60(-0.579–1.794)	0.30
**CC**	12(4/8)	1.11 (0.057–2.165)	**0.04**

*Bold text denotes significant p values (p<0.05).

^a^ β coefficients adjusted by age, contraceptive method and HPV-genotype

Shannon diversity index is expressed in bit units.

#### β-diversity

**Fig 4:**
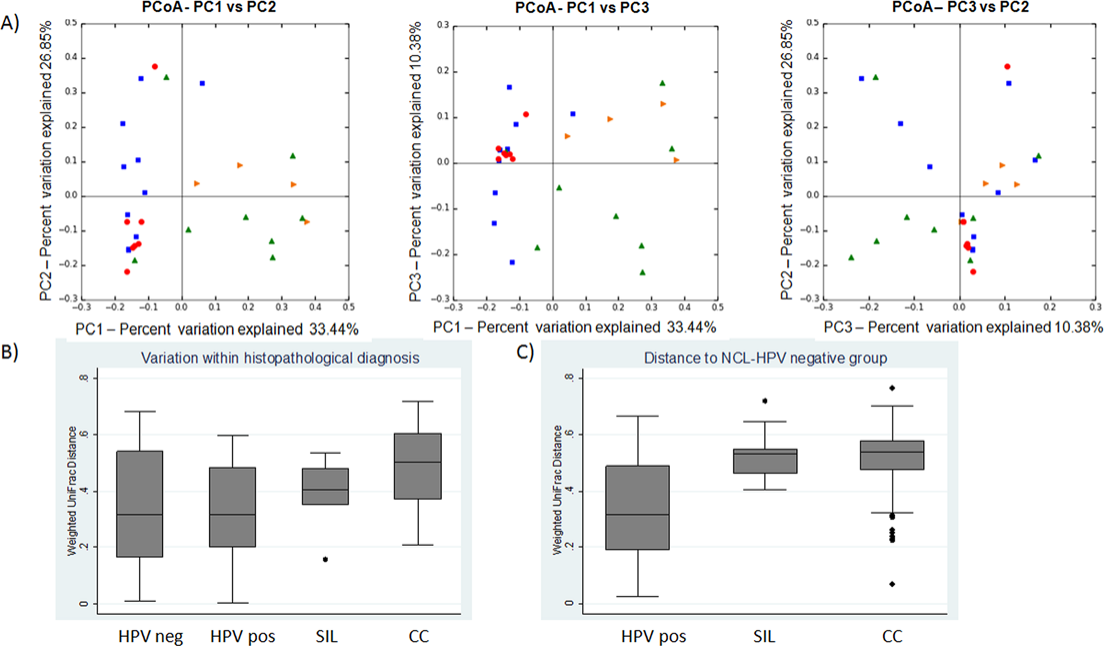
Beta-diversity of microbial communities by histopathological diagnosis. **A**. PCoA profile of the histopathological diagnosis displayed with weighted UniFrac distances. Each figure represents one sample colored according to its histopathological diagnosis. Red circles represent HPV-negative NCL samples; blue squares represent HPV-positive NCL samples; orange triangles represent SIL and green triangles represent CC. **A1**. Principal component (PC)-1 accounted for 33.44% of the variation in the composition of the microbiota due to the presence of *Sneathia spp*. and *Fusobacterium spp*. **A2**. PC-2 accounted for 26.85% of the variation inthe composition of the microbiota due to the presence of *Bifidobacteria spp*. and *Pseudomonas spp*. **A3.** PC-3 accounted for 10.38% of the variation in the composition of the microbiota due to the presence of *Lactobacillus spp*. and *Streptococcus spp*. **B. B1**. Variation of weighted UniFrac distances within each histological diagnosis group. **B2**. Variation of weighted UniFrac distances compared with -HPV-negativeNCL.

The PCoA results consistently showed that the cervical microbiome is notably different in every stage of the natural history of CC (**Fig 4A**). The three PCs where plotted in 2D for pairwise comparison; PC1 accounted for 33.44% of the variance between samples; PC2 for 26.85% and PC3 for 10.38%. The presence of *Fusobacterium spp*., *Sneathia spp*. and *Megasphaera spp*. was related to PC1. The presence of *Bifidobacteria spp*. and *Pseudomonas spp*. was related to PC2. The absence of *Pseudomonas spp*., *Fusobacterium spp*. and the presence of bacteria from the *Bifidobacteriaceae* family were related to PC3, according to the calculated factor loading matrix (**Table A in S1 File**).

When plotting PC1 vs PC2, we could see how the majority of the HPV-negative NCL samples clustered together in the third quadrant and none were in the first and forth quadrants, where CC and SIL samples were present. The plot of PC1 vs PC3 showed how all HPV-negatives NCL samples clustered together on the third quadrant, while 50% of the CC samples were only in the fourth quadrant, pointing to the presence of *Sneathia spp*. and *Fusobacterium spp*. and the absence of organisms from the *Bifidobacteriaceae* family. All SIL samples clustered in the first quadrant, pointing to the presence of *Fusobacterium spp*, *Sneathia spp* and *Megasphaera spp*. and a certain proportion of bacteria from the *Bifidobacteriaceae* family in this group.

When beta diversity was evaluated within each histological diagnosis group, the CC samples showed the highest variation within the groups (**Fig 4B1**, Kruskal–Wallis, p<0.0006) and the largest distance compared to HPV-negative NCL samples (**Fig 4B2**, U Mann-Whitney, p<0.00001).

#### Cervical expression of IL-4, IL-6, IL-10, TGF-β1, TNF-α and IFN-γ mRNA according to histopathological diagnosis

**Fig 5:**
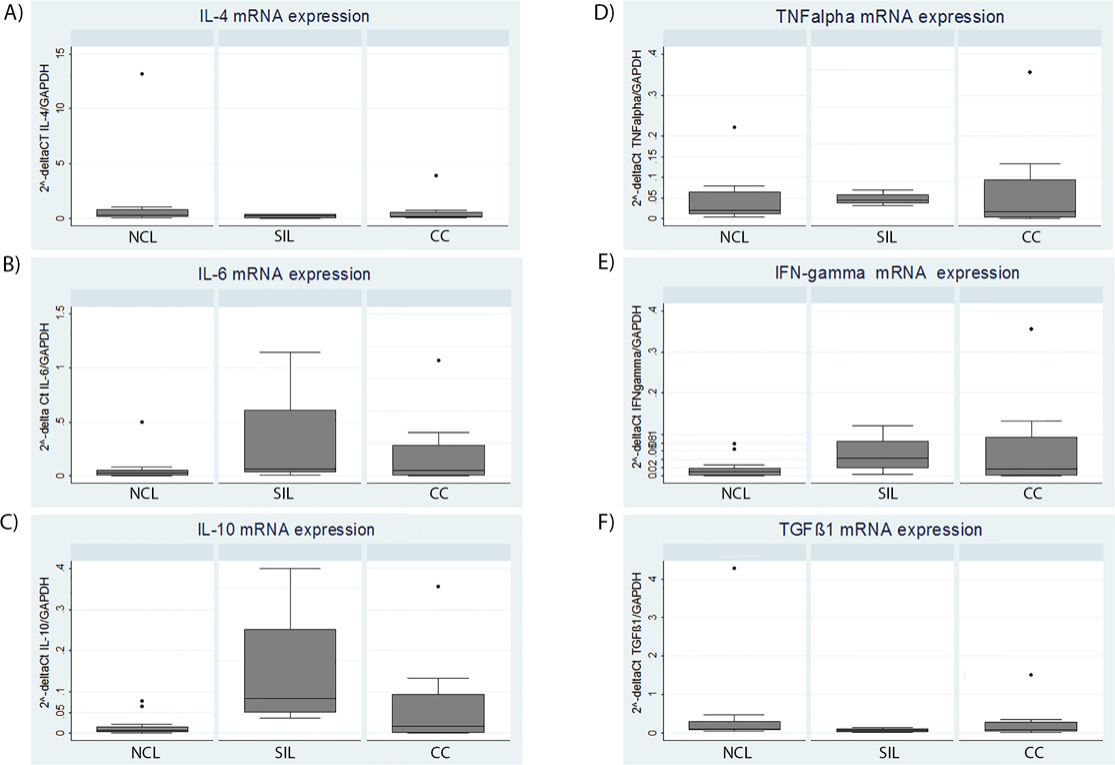
Cervical cytokine mRNA levels normalized to GAPDH, in NCL versus SIL and in NCL versus CC. (A) IL-4, (B) IL-6, (C) IL-10, (D) TNFα, (E) IFN-γ, (F) TGF-β1. * p value for the Mann-Whitney test (p = 0.04).

Cervical expression levels of IL-4, IL-6, IL-10, TGF-β1, TNF-α and IFN-γ mRNA from NCL vs SIL and NCL vs CC cases normalized to GAPDH mRNA are shown in **Fig 5**. Even though discrete differences can be observed between cervical expression levels of IL-4, IL-6, TGF-β1, TNF-α and IFN-γ mRNA normalized to GAPDH mRNA, only the median levels of IL-10 mRNA relative to GAPDH were higher in the SIL cases than in the NCL ones. The difference was statistically significant (p = 0.04).

#### Evaluation across community state type clusters

**Fig 6:**
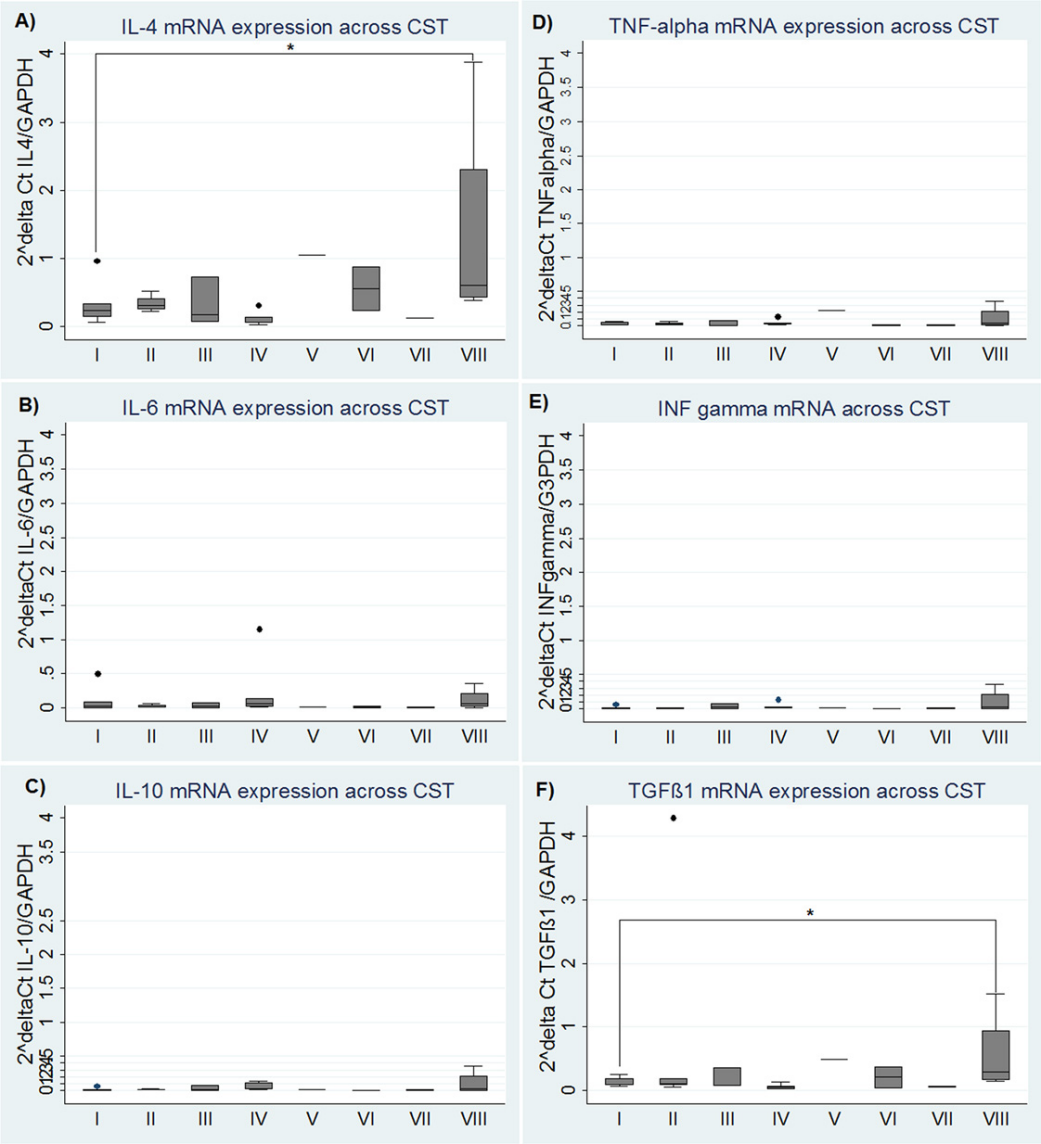
Cervical cytokine mRNA levels normalized to GAPDH per community state type (CST). (A) IL-4, (B) IL-6, (C) IL-10, (D) TNFα, (E) IFN-γ, (F) TGF-β1. *p value for Kruskal Wallis test (p≤0.05).

**Fig 7:**
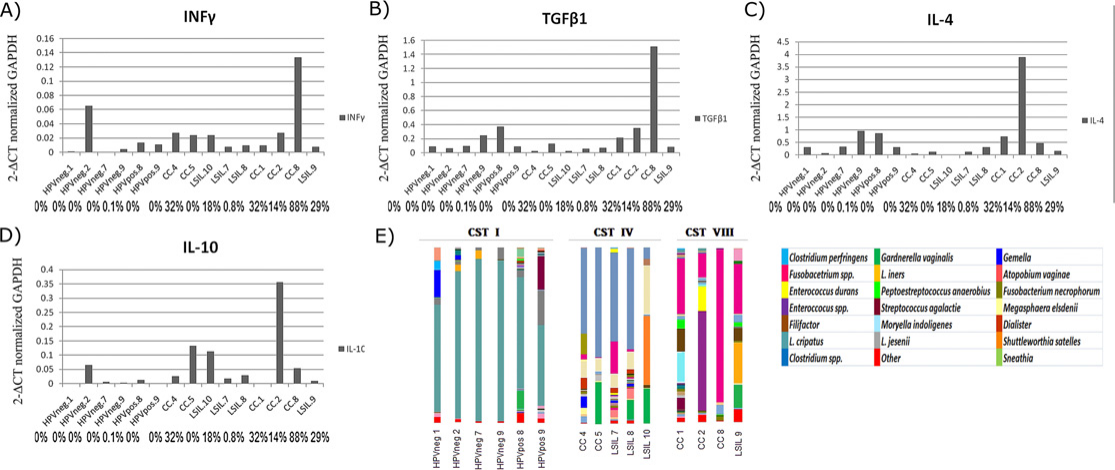
INF-γ, IL-4, TGF-β1 and IL-10 expression level per sample in community state type CST I, CST IV and CSTVIII and microbiome composition. Cervical expression level of INF-γ (A), TGF-β1 (B), IL-4 (C) and IL-10 (D) normalized with GAPDH gen. (E) Microbiome composition in relative abundance.

When cervical expression of IL-4, IL-6, IL-10, TGF-β1, TNF-α and IFN-γ mRNA was evaluated across CST clusters, we found higher median cervical levels of IL-4 and TGF-β1 mRNA relative to GAPDH in CST VIII dominated by *Fusobacterium spp*., and the difference was statistically significant (p = 0.05 and p = 0.04, respectively) (**Fig 6**).To see if these findings could be explained mainly by the presence of *Fusobacterium spp*., the microbial composition of samples from CST I, CST IV and CST VIII were compared with their respective IL-4, TGF-β1 and INF-γ expression level (**Fig 7**). The highest level of IL-4 was found in sample CC2, whereas the highest level of TGF-β1 was found in sample CC8, which is composed of 87.95% *Fusobacterium spp*.

Finally, when we analyzed the direct correlation between the Shannon diversity index and the PD whole tree and the cervical expression of IL-4, IL-6, IL-10, TGF-β1, TNF-α and IFN-γ mRNA, we did not find any correlation.

## Discussion

Several factors are implicated in the development of CC, including bacterial co-infection [49].The main findings of this study showed that the cervical microbiome is notably different in all stages of the natural history of CC. Surprisingly and in spite of the small sample size, we found higher median cervical levels of IL-4 and TGF-β1 mRNA in CST VIII, dominated by *Fusobacterium spp.*. As expected, we found high abundance of *Lactobacillus spp*. in the cervix of HPV-negative women without lesions, and *L*. *crispatus* was the most abundant species. In the cervix of HPV-infected women without lesions, the most abundant species was *L*. *iners*. Two of the CST detected in this study (CST I and II, dominated by *L*. *crispatus* and *L*. *iners*, respectively), which were described only in HPV-negative and -positive women with NCL, were similar to those reported by Ravel et al. and Smith et al. [50, 32]. Moreover, CST V, dominated by *Gardnerella vaginalis*, was found in a healthy Hispanic population in Smith’s study [32]. Likewise, Huang et al. reported similar CSTs in the cervix of their control group (non-pregnant Korean women)[30].

Our findings agree with the latest studies of HPV-infected women’s vaginal microbiome (**Table 6**). Previous reports on vaginal microbiota state that bacterial diversity among Chinese and Korean HPV-positive women is more complex than among healthy women [34, 50]. Longitudinal studies of vaginal microbiota have shown that *L*. *crispatus* is more beneficial than *L*. *iners*. [51]. *L*. *iners* has been found more often in women with vaginal dysbiosis by HIV, HPV and HSV-2 than *L*. *crispatus* [52]. Unlike *L*. *iners*, *L*. *crispatus* produces hydrogen peroxide. This could explain why *L iners* is found in a higher proportion than *L*. *crispatus* among HPV-infected women, since hydrogen peroxide has been recognized as critical in maintaining a healthy vaginal microbiota [53].

**Table 6:** Comparison of microbiota composition between different studies and the present study.

HPV status	Population	Vagina CST	Cervix CST	Pilot study	Reference
HPV-NCL	Caucasian; Hispanic; Japanese; Asiatic	*L*. *iners; L*. *crispatus; L*. *gasseri; Gardnerella vaginalis; Prevotella; Sneathia (1*,*2*,*3)*	*L*. *iners; L*. *crispatus; L*. *gasseri; Gardnerella vaginalis (4)*	*L*. *iners; L*. *crispatus; Gardnerella vaginalis; Streptococcus agalactiae*	(1).Zou X, et al. 2004; (2) Ravel J; et al. 2011; (3) Gager P, et al. 2012; (4) Smith BC, et al 2012
HPV+ NCL	Korean	*Sneathia; Fusobacterium spp*.*; Dialister (5)*	*??*	*L*. *iners; L*. *crispatus; Gardnerella vaginalis; Pseudomonas oleovorans*	(5) Gao W, et al 2013
SIL	Chinese	*Lachnospiraceae*	*Atopobium vaginae; Gardnerella vaginalis*	*Sneathia spp*.*; Fusobacterium spp*.*; Shuttleworthia satelles; Megasphaera elsdenii*	(6) Lee JE, et al. 2013 (7) Oh HY, et al. 2015
CC	Mexican	*??*		*Fusobacterium necrophorum; Fusobacterium spp*.*; Sneathia spp*.*; Enterobacterium; Streptoccoccus agalactiae*	

*Sneathia spp*., *Megasphaera elsdenii* and *Shuttleworhia satelles* were most representative in the SIL cases. Similarly to our findings, Fusobacteria, including *Sneathia spp*., were previously identified as a possible microbiological marker associated with the HPV-infection in a Korean twin cohort [54]. Bacteria of the genus *Sneathia* are arising as possible pathogens of the female reproductive tract. *Sneathia spp*., previously named *Leptotrichia amnionii*, can be part of the normal microbiota of the genitourinary tracts of men and women [55]. A correlation between colonization with *Sneathia amnii* (“*L*. *amnionii*”) and CC in HPV-positive subjects has been documented [56]. In our study, we found that *Sneathia spp*. was the most abundant species in the cervix of women with SIL and it was less abundant in patients with CC. Taken together, these results indicate that the presence of *Sneathia spp*. is a characteristic trait of patients with HPV-positive SIL.

*Megasphaera elsdenii* and *Shuttleworthia satelles* have not been previously reported in women with SIL. This was somewhat unexpected as *Megasphaera type 1* and *Sneathia amnionii* are considered good predictors of BV [57], which is a classical dysbiosis associated with abnormalities in cervical smears [58]. Little is known about *Shuttleworthia satelles*. The only finding is that it has been isolated from the human oral cavity and from one patient with endocarditis and prosthetic valve infection [59].

Remarkably, our findings on the late stages of CC showed that *Fusobacterium spp*. is significantly more abundant than in the early stages (HPV-negative or -positive NCL),and *F*. *necrophorum* was only observed in the CC group. In agreement with our results, Fusobacteria and *Atopobium vaginae* were also predominant in the vaginal microbiota of Korean women with high risk of cervical intraepithelial neoplasia (CIN) [60].

*Fusobacterium* species are anaerobic Gram-negative bacteria that are part of the normal flora in the human mouth and the gut mucosa [61]. On the other hand, some species of *Fusobacterium spp*. have been recognized as opportunistic pathogens in inflammatory diseases in both mouth (periodontitis) and gut (bowel disease) [62]. Furthermore, *Fusobacterium spp*., have been linked to colorectal cancer [63, 64]. Molecular studies have confirmed these results and a signature of virulence genes from the microbiota has been identified [65–67].

Some studies have shown the molecular mechanisms by which *Fusobacterium spp*. induces its pathogenic effects. *Fusobacterium nucleatum* promotes colorectal carcinogenesis by modulating the E-cadherin/β-catenin signaling via its FadA adhesin, thus modifying the tumor-immune microenvironment [68, 69]. The process is as follows: FadA binds to E-cadherin/β-catenin; E-cadherin is phosphorylated on the membrane and together with FadA, they are internalized, and beta-catenin is accumulated in the cytoplasm and translocated, and results in activation of the transcription factor NF-kB in the nuclei [68]. Moreover, fadA gene levels in the colorectal epithelium from colorectal carcinoma patients are higher than in normal patients [68]. Given that *Fusobacterium spp*. appear in high proportion in CC, it is possible that the FadA gene levels could be overexpressed in cervical cancer patients. Likewise, previous reports demonstrate an abnormal distribution of tumor-suppressor E-cadherin, which functions through beta-catenin, in different histological types of CC [70–72].

In terms of the aim of our study, finding out whether there is a relationship between cervical microbiota and a specific cytokine profile in women with different stages of CC, we found significant differences between CST VIII (conformed by SIL and CC cases) and high levels of cervical IL-4 and TGF-β1 mRNA. Several reports, including some from our laboratory, have shown high mRNA levels of TGF-β1, IL-4, and IL-10 in cervical biopsies of patients with SIL and CC [25, 34]. Similarly, high systemic levels of these cytokines have been reported in patients with CC [13]. It has been reported that oral infection by *Fusobacterium* enhances the systemic level of IL-4 [73]. Thus, a *Fusobacterium* infection could play a key role in the development of an immunosuppressive microenvironment characterized by anti-inflammatory cytokines (Th2 cytokine profile), such as IL-4 and TGF-β1, in HPV-transformed cells from the uterine cervix.

Likewise, it was demonstrated in a recent study that the microbiota regulates Th2 immunity through the induction of type 3 RORγτ+ Tregs and Th17, acting as a key element to balance the immune responses of the epithelial cells [74]. T-lymphocyte infiltration of the cervical mucosa increases according to the degree of CC malignancy and correlates with FoxP3+ lymphocyte infiltration in the later stages [75]. CD4+CD25+Foxp3+ Tregs play an important role in the pathogenesis of cervical carcinoma [76]. Therefore, the presence of some members of the cervical microbiota, such as *Fusobacterium spp*., may induce Th2 immunity through the RORγτ+ Treg and Th17 cells, present in the cervical epithelium [77]. The transdifferentiation of Th17 into Tregs cells was recently illustrated: It was demonstrated by changes in their transcriptional profile and the acquisition of potent regulatory capacity. Different transcriptional profiles of pre- and post-conversion Th17 cells revealed a role for canonical TGF-β signaling [78].

Another Th2 cytokine implicated in cervical immunosuppression in CC patients is IL-10 [24]. In this study, in spite of the presence of statistically significant high IL-10 mRNA levels in SIL, we did not find any association with CST VIII. The source of high cervical production of IL-10 by tumor cells, keratinocytes, macrophages and Langerhans cells in SIL and CC cases has been demonstrated by an immunohistochemical analysis [24, 79]. Several factors contribute to a high cervical production of IL-10 and TGF-β1 in HPV-transformed cells, including HPV proteins such as E2 [80] and HPV E6/E7 oncoproteins [81], which induce a transcriptional up-regulation of IL-10 and TGF-β1 expression [34].

On the other hand, cervical high levels mRNA of IFN-γ had been found in CC cases [25, 82]; in despite of this, evaluation of serum from patients with cervical lesions and from HPV-transformed cells, exert a strong immunosuppressive effect [25–27, 82]. Additionally, IFN-γ production favors the presence of iNKC in CIN patients and in HPV oncogene–driven hyperplasia and this play an important role in suppressing cellular immunity [83, 84]. This evidence supports the idea that several processes collaborate to maintain an immunosuppressive microenvironment and favor CC development.

The evidence reported in literature indicates that the expression levels of IFN-γ differ at local and systemic level in women with CC[13, 25]. Recently, we reported that women with minor allele homozygote genotype of the polymorphism -1615C>T of IFN-γ have a significant negative association with CC and these patients present lower serum levels than those observed in HPV-positive control patients [13]. Furthermore, the IFN-γ polymorphism studied extensively is +874T>A (rs2430561) and A/A genotype has been associated with low producer phenotype of IFN-γ [85]. Since mRNA IFN-γ levels are variable at cervical level in the natural history of CC [86], so that IFN-γ would be exercising two functions in the natural history of CC, in the initial phases exert an antiviral effect to control the infection and subsequent stages an immunosuppressive effect and in consequence a CC progression.

**Fig 8:**
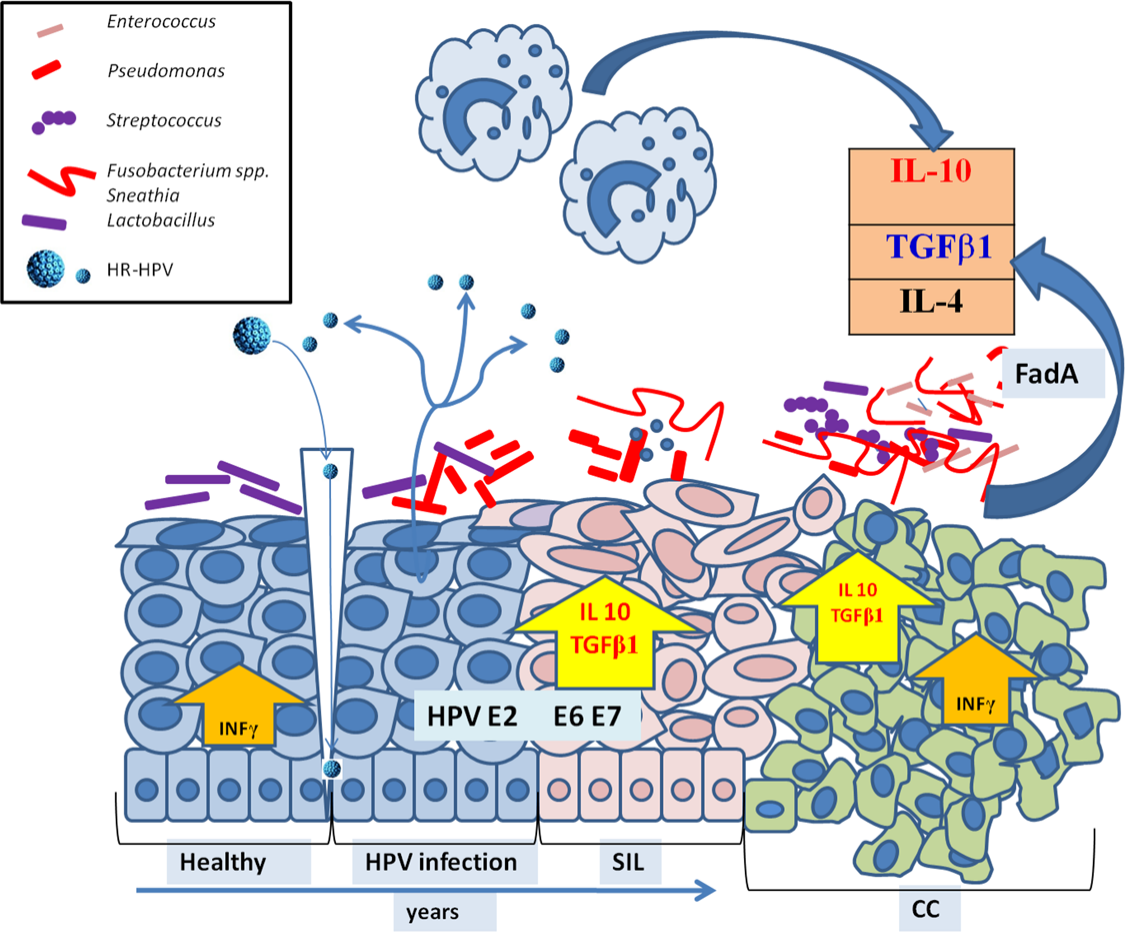
Suggested mechanism of microbiome changes during immunosuppression development. The cervical epithelium is represented in each stage of CC as departing from a normal epithelium (left) and its longitudinal change when a HR-HPV infects it and it progresses to SIL and CC. Microbiome composition and diversity is depicted according to the main bacteria per stage. *Lactobacillus* are represented as purple rectangles, *Pseudomonas oleovorans* as red rectangles, *Fusobacterium* and *Sneathia* as shaped rods, *Streptococcus agalactie*as purple circles, and HPV as blue circles. After infection takes place, the microbiome changes and its diversity increases. HPV proteins E2, E6 and E7 enhance IL-10 expression and macrophages type 2 presence. The latter is also enhanced by TGFβ-1, which is in turn stimulated by the microbiota present. The diversity of the microbiota increases, through its toxins (FadA from *Fusobacterium spp*.), which disturb tight junctions and promote a metastasis similar to colon carcinoma.

After everything that has been discussed, we arrive to the following possible mechanisms leading to cervical cancer progression, as illustrated in **Fig 8**. After a HR-HPV infection in a normal epithelium, the microbiome switches its composition from being dominated by *L*. *crispatus* to *L*. *iners*. With the progression to a SIL, the diversity of the microbiota increases with the appearance of *Sneathia* and other *Fusobacterium spp*. When CC appears, *Fusobacterium necrophorum* is present. This change in composition and diversity could be explained by the immunosuppressive microenvironment triggered by the viral infection, and it contributes to maintain a positive feed-back loop between the cytokine profile and the cervical microbiota.

## Conclusions

In conclusion, we suggest that some members of the cervical microbiota are possible modifiers of the cytokine profile of the cervical microenvironment during the development of SIL and CC. Currently, a Mexican cohort study is being carried out to support these findings. Further studies are needed to delve into the mechanism used by bacteria to promote cervix immunosuppression in CC. Evidence is accumulating and it points toward a major role of the microbiota in the immune system modulation of the female genital tract [31].

We consider that this opens new trends for understanding the role of *Fusobacterium spp*. in cervical carcinogenesis. *Fusobacterium spp*. either contributes by shifting Th1 immunity to Th2 or by a direct effect on the E-cadherin/β-catenin signaling pathway on cervical HPV-transformed cells. The findings reported here may allow, after validation, to build up new diagnostic strategies with microbiological markers for patient identification during the early stages of cervical cancer.

## Supporting Information

S1 File **Fig A**, Alpha diversity rarefaction curves **Table A,** Factor loading matrix for cervical microbiota composition.
(PDF)

S1 Table Proportions of each OTU present in samples. (XLSX)
