# Supplementary material for: Cervical Microbiome and Cytokine Profile at Various Stages of Cervical Cancer: A Pilot Study
Source: PLoS One. 2016 Apr 26;11(4):e0153274. doi: 10.1371/journal.pone.0153274 (PMC4846060; doi:10.1371/journal.pone.0153274)
Supplement: S1 File — Fig A, Alpha diversity rarefaction curves Table A, Factor loading matrix for cervical microbiota composition. (PDF) [file pone.0153274.s001.pdf]

Figure A)

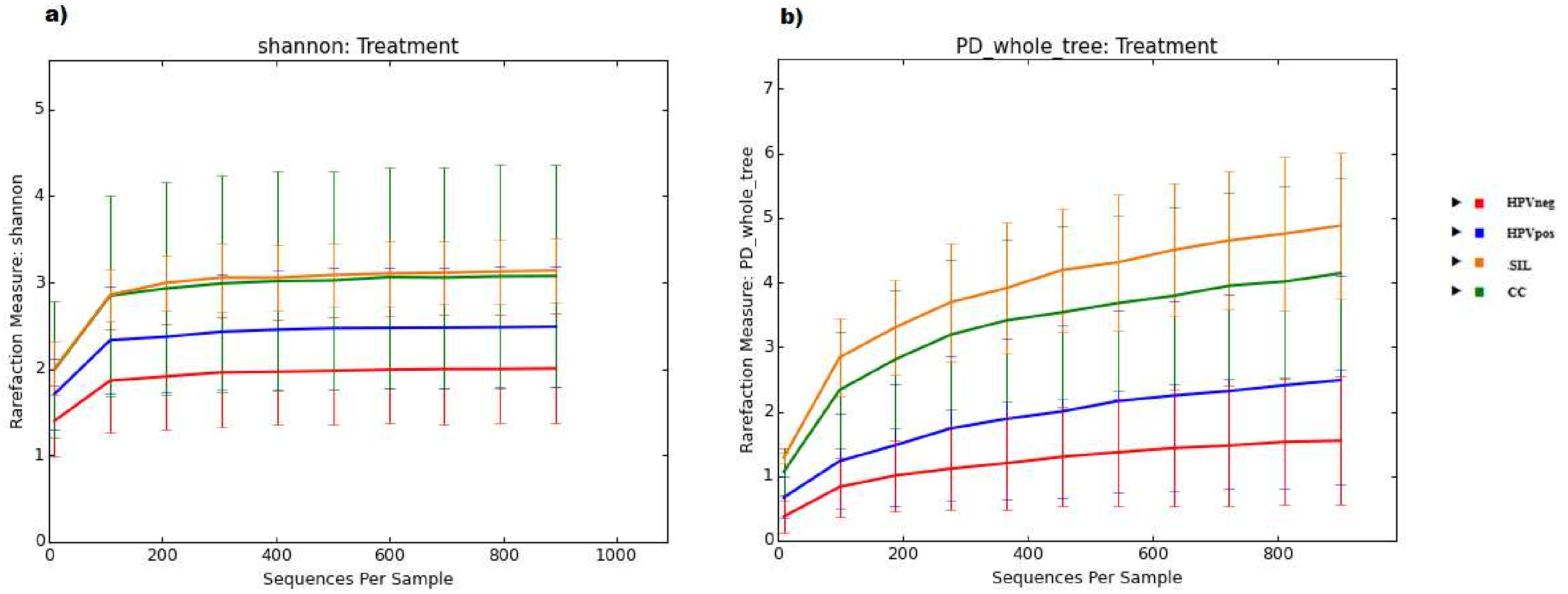

Table B)

Factor-loading matrix for Cervical Microbiota Composition			
**Taxon**	**PC1**	**PC2**	**PC3**
Lactobacillus spp.	-0.14	-0.07	0.02
Bifidobacteriaceae spp.	0.03	0.23	0.11
Fusobacterium spp.	0.25	-0.12	-0.16
Pseudomonas spp.	-0.15	0.19	-0.12
Sneathia spp.	0.34	0.01	0.09
Streptococcus spp.	-0.14	-0.18	0.02
Mycoplasma spp.	0.09	0.00	0.02
Enterococcus spp.	0.02	-0.10	-0.05
Megasphaera spp.	0.28	0.04	0.09
Dialister spp	0.16	0.12	0.03
